# Artificial intelligence improves survival prediction in patients with brain metastases submitted to radiosurgery

**DOI:** 10.1007/s10143-025-04051-6

**Published:** 2026-02-07

**Authors:** Eliseu Becco Neto, João Paulo Mota Telles, Larissa Zaira Rafael Rolim, Francisco de Assis de Souza Filho, Vinicius Costa Becco de Souza, Letícia Costa Becco de Souza, Helvécio Neves Feitosa Filho, Rodrigo Becco de Souza, Dhiego Chaves de Almeida Bastos, Sujit Prabhu, Eberval Gadelha Figueiredo

**Affiliations:** 1 Division of Neurosurgery, Department of Neurology, University of São Paulo, São Paulo, Brazil; 2 Strategic Center of Excellence in Water and Drought Policies, Federal University of Ceará, Ceará, Brazil; 3 Faculty of Medicine, Federal University of Ceará, Ceará, Brazil; 4 Medical School, Christus University, Ceará, Brazil; 5 Faculty of Medicine, University of Fortaleza, Ceará, Brazil; 6 Neurosurgery Department, Cleveland Clinic, Cleveland, OH USA; 7 Department of Neurosurgery, The University of Texas MD Anderson Cancer Center, Houston, TX USA; 8 Hospital das Clínicas da Faculdade de Medicina da Universidade de São Paulo (HCFMUSP), Av Dr Eneas Carvalho de Aguiar, 255, São Paulo, Brazil

**Keywords:** Brain neoplasms, Neoplasm metastasis, Radiosurgery, Artificial intelligence

## Abstract

Stereotactic radiosurgery (SRS) is effective for non-small cell lung cancer (NSCLC) brain metastases in deep or eloquent brain regions. Identifying predictors of treatment failure is crucial. Artificial intelligence (AI) models may improve prediction, but data on NSCLC BM are scarce. A retrospective study analyzed NSCLC patients with single brain metastases treated with SRS (Elekta Gamma Knife) from 2010 to 2015, with up to 10 years of follow-up. Clinical, radiological, and histological data were collected. Kaplan-Meier and Cox proportional hazards models assessed survival. Decision tree and random forest (RF) models predicted treatment failure, with feature importance analyzed. Among 133 patients (mean age 61.6, 56.4% male), most tumors were grade 1 (56.4%) and in the right hemisphere (60.2%). The mean tumor volume was 1.84 cm³. Decision trees identified metastasis volume and location as key predictors (AUC = 0.85). RF models improved prediction (AUC = 0.92). Tumor volume, diameter, and age were major predictors. AI models effectively identified patients at risk of treatment failure. A random forest based artificial intelligence model presented an excellent predictive ability for stereotactic radiosurgery success/failure in a population with NSCLC brain metastases, with an area under curve of 0.92. This predictive ability was superior to a decision tree or a simple diameter-to-volume ratio.

## Introduction

Treatment and understanding of brain metastases are very important, as they correspond to 50% of intracranial tumors [1–5]. A significant proportion of patients with non-small cell lung cancer (NSCLC) will develop metastases. When untreated, these patients may experience survival expectancies as low as 1–2 months [6, 7].

Stereotactic radiosurgery is a safe and effective treatment for these patients, particularly for metastases otherwise unfit for surgical treatment, such as those in profound or eloquent areas [8–10]. It is important to identify predictors of treatment failure/success to identify patients most suited for each treatment.

Artificial intelligence models provide an excellent means to classifying and identifying individuals as more prone to a favorable outcome. However, data on this subject regarding NSCLC central nervous system metastases are scarce. This article aims to identify predictors of radiotherapy success/failure in these patients.

## Methods

This was a retrospective study of patients with NSCLC BM who had a single metastasis treated using stereotactic radiosurgery (SRS), (Elekta Gamma Knife Perfexion System) between 01/01/2010 and 12/31/2015. Patients were followed for up to 10 years after SRS. The study was approved by the ethics review board (# 2.579.524, 04/04/2018). Previous publications from our group report detailed data and outcomes of this population [11, 12].

In order to build the predictive models of this analysis, the following information were collected: clinical, epidemiological, radiological, and histological data. Tumor metrics, such as volume, diameter, tumor grade, homogeneity in imaging studies, and radiation dose levels. Location-specific tumor data were also structured for analysis, with brain regions categorized by anatomical location.

Tumor volume (in cm³) was measured, and the corresponding diameter was calculated using the formula for the diameter of a sphere:

$$\:D=2\left(\frac{3\cdot\:V}{4\pi\:}\right)^\frac13$$

where V is the tumor volume and D is the diameter. These derived variables were used in survival modeling and predictive analyses.

### Statistical analyses

The Kaplan-Meier (KM) estimator was employed to model survival probabilities over time. The analysis stratified patients based on tumor laterality and laterality-tentorium combinations to explore differences in survival outcomes. Differences between groups were assessed using the log-rank test. The Cox proportional hazards model was applied to assess the combined effects of multiple predictors on survival time. The likelihood ratio, Wald, and log-rank tests were used to assess model significance. The concordance index was computed to evaluate model performance.

## Artificial intelligence models

In this study, a decision tree models was applied to classify treatment outcomes based on key variables and complemented this approach with a Random Forest model to address the limitations inherent to individual trees. The decision tree algorithm, as proposed by Breiman et al. (1984), recursively segments the dataset into nodes using impurity criteria, with splits chosen to minimize impurity at each node. A pruning technique was applied to prevent overfitting. The decision tree was built using the rpart package in R (Therneau and Atkinson, 2015), with predictor variables such as final volume and brain location. Hyperparameter tuning—particularly for the complexity parameter (cp.)—was performed via grid search combined with cross-validation using the caret package.

To mitigate issues like high variance and potential overfitting associated with single decision trees, we employed the Random Forest (RF) algorithm (Breiman, 2001). RF is an ensemble method that constructs multiple decision trees from random subsets of the data and variables, aggregating predictions through majority voting (for classification) or averaging (for regression). Additionally, the use of out-of-bag (OOB) estimates allowed us to evaluate model performance without requiring a separate test set.

Hyperparameter tuning for the Random Forest was also carried out using a grid search with cross-validation implemented in caret. The parameter grid included mtry (the number of variables sampled at each split), splitrule (the splitting criterion, such as “gini” or “extratrees”), and min.node.size (the minimum size of terminal nodes). This systematic approach enabled us to identify the best combination of parameters based on predictive performance during validation. The final RF model was fitted using the caret package with the optimal mtry value and a fixed number of trees (ntree = 100).

Furthermore, variable importance was assessed using metrics such as mean decrease in accuracy and mean decrease in Gini impurity. Complementary analyses, including ROC curves and importance plots, were performed to validate both model performance and interpretability.

## Results

### Patient characteristics

**Fig. 1:**
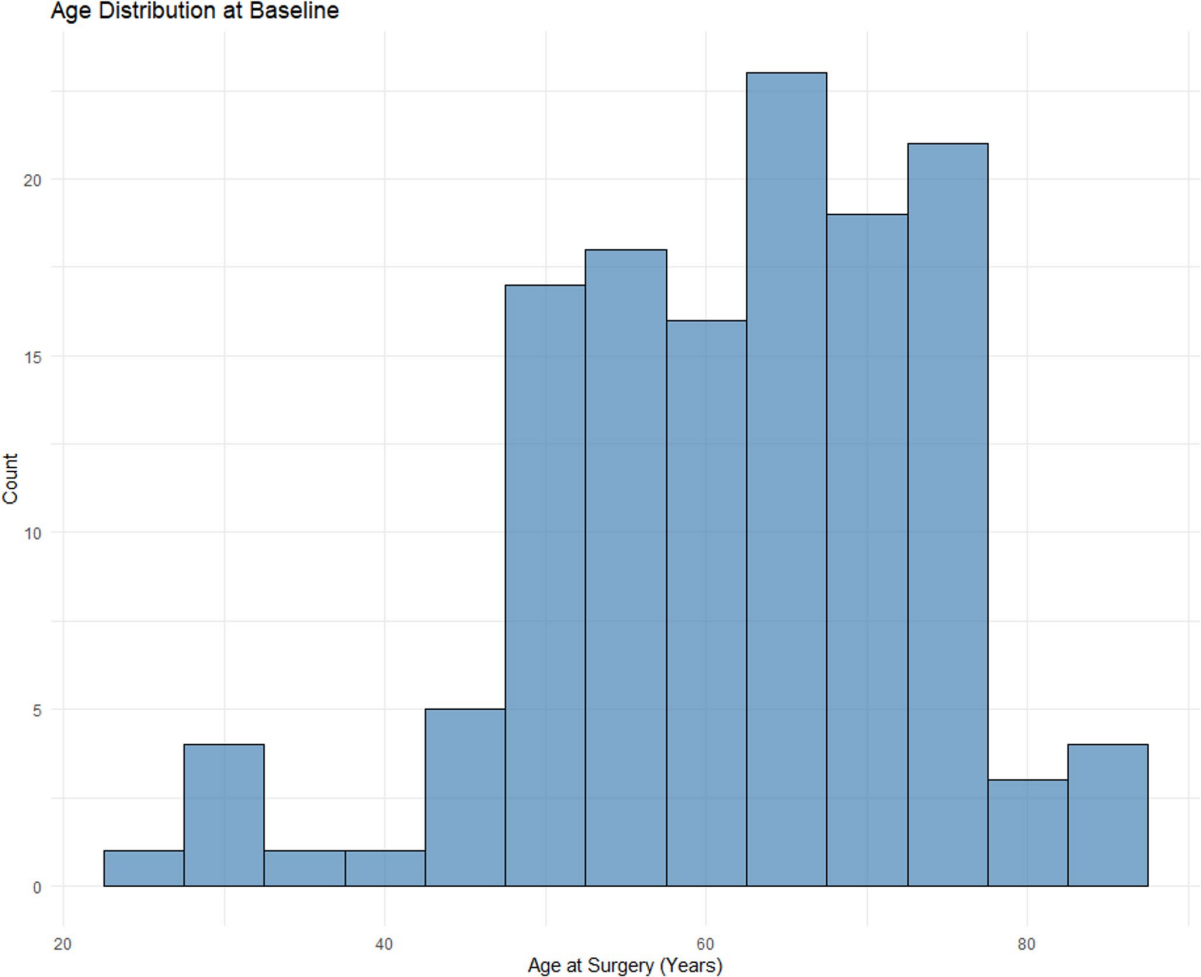
Age distribution at baseline

The dataset comprised 133 patients (Table 1) diagnosed with brain metastases, with an average age at surgery of 61.6 years (SD = 12.1) (Fig. 1). The sample was predominantly male (56.4%, *n* = 75), with females accounting for 43.6% (*n* = 58). Preoperative functional status, assessed by the Karnofsky Performance Score (KPS), had a mean value of 86.7 (SD = 10.9).

**Table 1:** Patient characteristics

Age		61.57 (12.08)
Female		58 (43.6)
KPS		86.69 (10.85)
Tumor Grade	1	75 (56.4)
	2	11 (8.3)
	3	43 (32.3)
	4	4 (3.0)
Side	Left	50 (37.6)
	Right	80 (60.2)
	Midline	2 (1.5)
	Other	1 (0.8)
Homogeneity		1.29 (0.46)
Dose (Gy)	10	1 (0.8)
	12	1 (0.8)
	14	2 (1.5)
	15	3 (2.3)
	16	1 (0.8)
	17	2 (1.5)
	18	19 (14.3)
	19	2 (1.5)
	20	91 (68.4)
	21	1 (0.8)
	22	7 (5.3)
	24	2 (1.5)
Location	Basal Ganglia	1 (0.8)
	Medulla	1 (0.8)
	Pons	3 (2.3)
	Cerebellum (Brachium Pontis)	1 (0.8)
	Cerebellum (Hemisphere)	20 (15.0)
	Cerebellum (Vermis)	2 (1.5)
	Frontal	54 (40.6)
	Insula	1 (0.8)
	Occipital	15 (11.3)
	Parietal	20 (15.0)
	Temporal	12 (9.0)
	Thalamus	3 (2.3)
Volume (cm^3^)		1.84 (2.68)
Diameter (cm)		1.26 (0.60)

Data are presented as mean ± standard deviation or frequency (%), as appropriate

*KPS* Karnofsky Performance Scale

Caption: Decision tree for prediction of treatment failure. Significant nodes include initial volume (Volume(i)), final volume (Volume(f)), and tumor location

Caption: Area under curve (AUC) comparison for three different predictors: decision tree, random forest, and diameter/volume ratio. The random forest had the best AUC (0.92), followed by the decision tree (0.85), and the diameter/volume ratio (0.72)

Tumor grades were distributed with 56.4% (*n* = 75) classified as grade 1, 32.3% (*n* = 43) as grade 3, 8.3% (*n* = 11) as grade 2, and 3.0% (*n* = 4) as grade 4. Tumor laterality showed that 60.2% (*n* = 80) were located on the right side of the brain, while 37.6% (*n* = 50) were on the left. A small percentage (2.3%, *n* = 3) had midline or other placements.

The mean tumor homogeneity score was 1.29 (SD = 0.46). Radiation doses varied significantly, with the most commonly administered dose being 20 Gy (68.4%, *n* = 91), followed by 18 Gy (14.3%, *n* = 19). Smaller proportions of patients received doses ranging from 10 Gy to 24 Gy.

In terms of tumor location, the frontal lobe was the most frequent site (40.6%, *n* = 54), followed by the parietal (15.0%, *n* = 20) and occipital lobes (11.3%, *n* = 15). The cerebellum (15.0%, *n* = 20) and temporal lobe (9.0%, *n* = 12) were other common sites. Less frequent sites included the thalamus (2.3%, *n* = 3), brain stem (2.3%, *n* = 3), and basal ganglia (0.8%, *n* = 1).

**Fig. 2:**
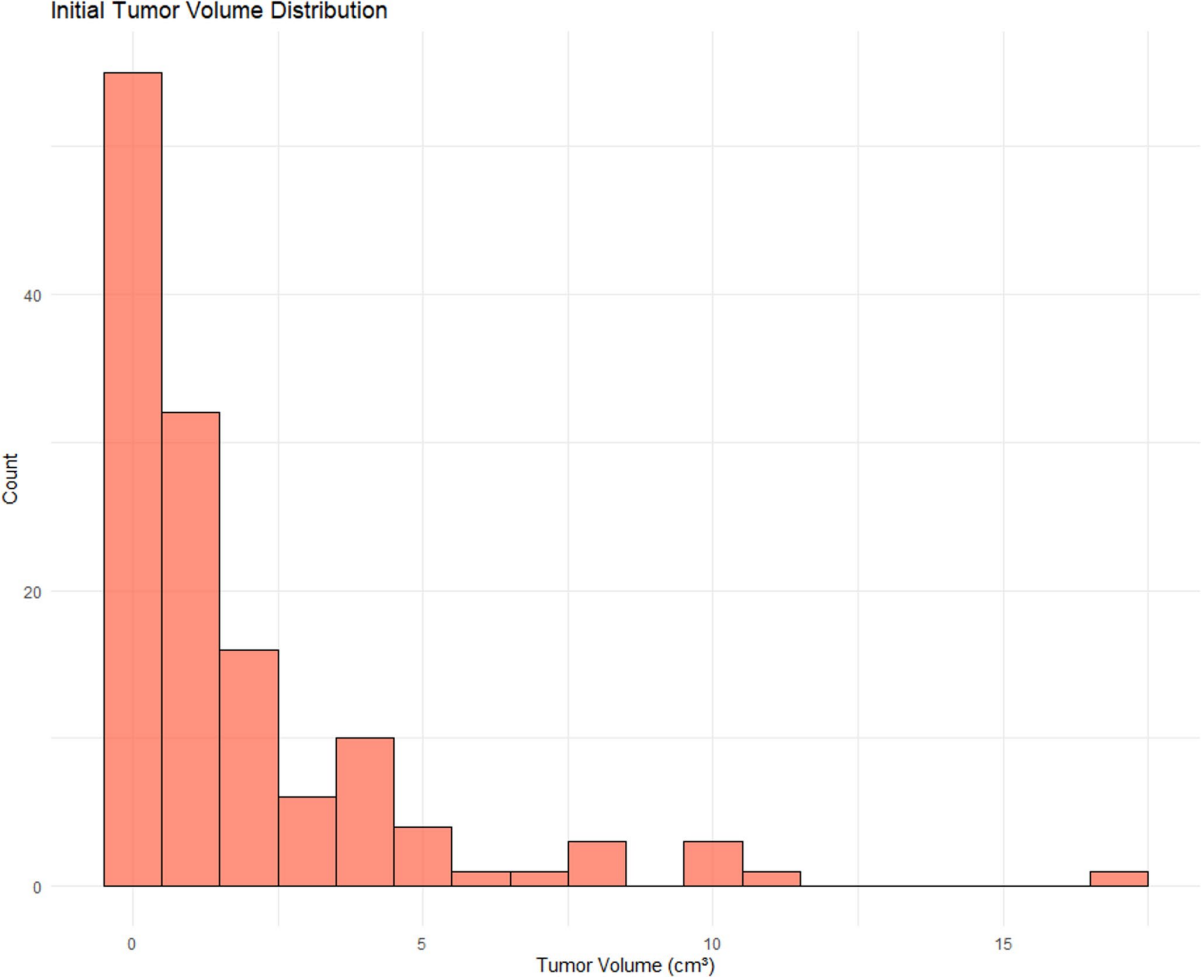
Tumor volume distribution

The initial tumor volume had a mean of 1.84 cm³ (SD = 2.68) (Fig. 2), while the mean initial tumor diameter was 1.26 cm (SD = 0.60).

### Treatment failure prediction using decision trees and random forests

**Fig. 3:**
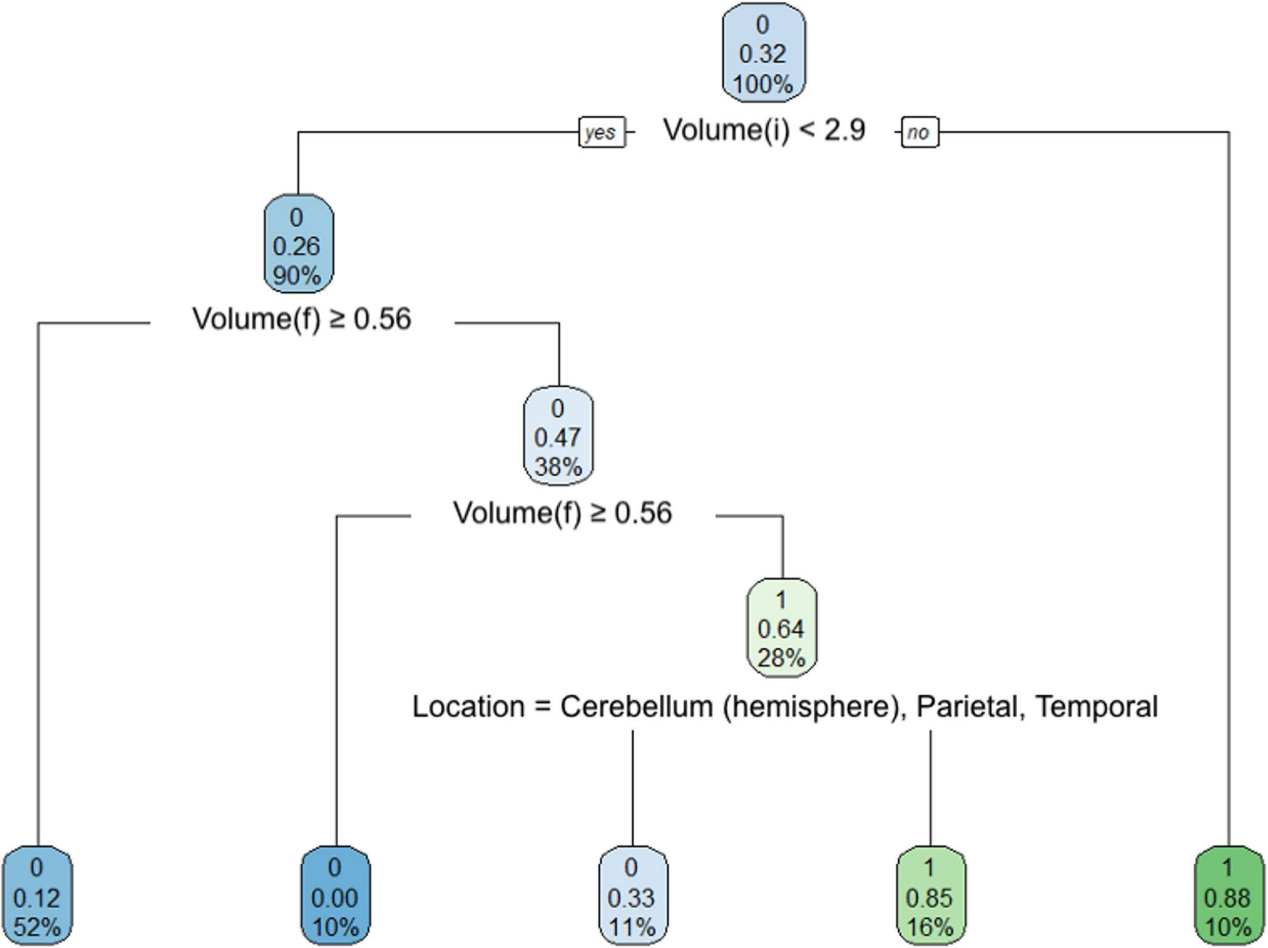
Decision tree for prediction of treatment failure. Significant nodes include initial volume (Volume(i)), final volume (Volume(f)), and tumor location

**Fig. 4:**
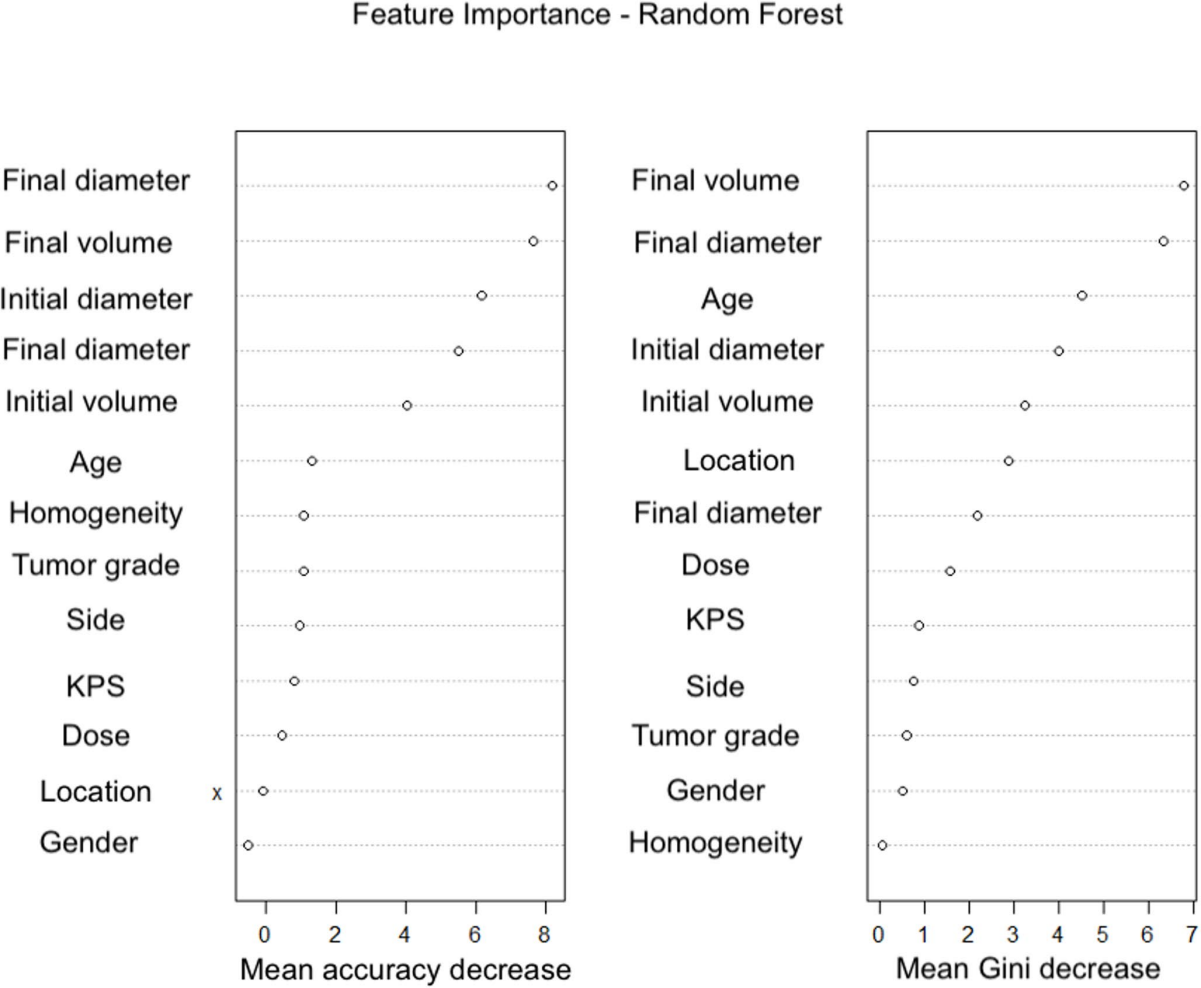
Random forest: feature importance

**Fig. 5:**
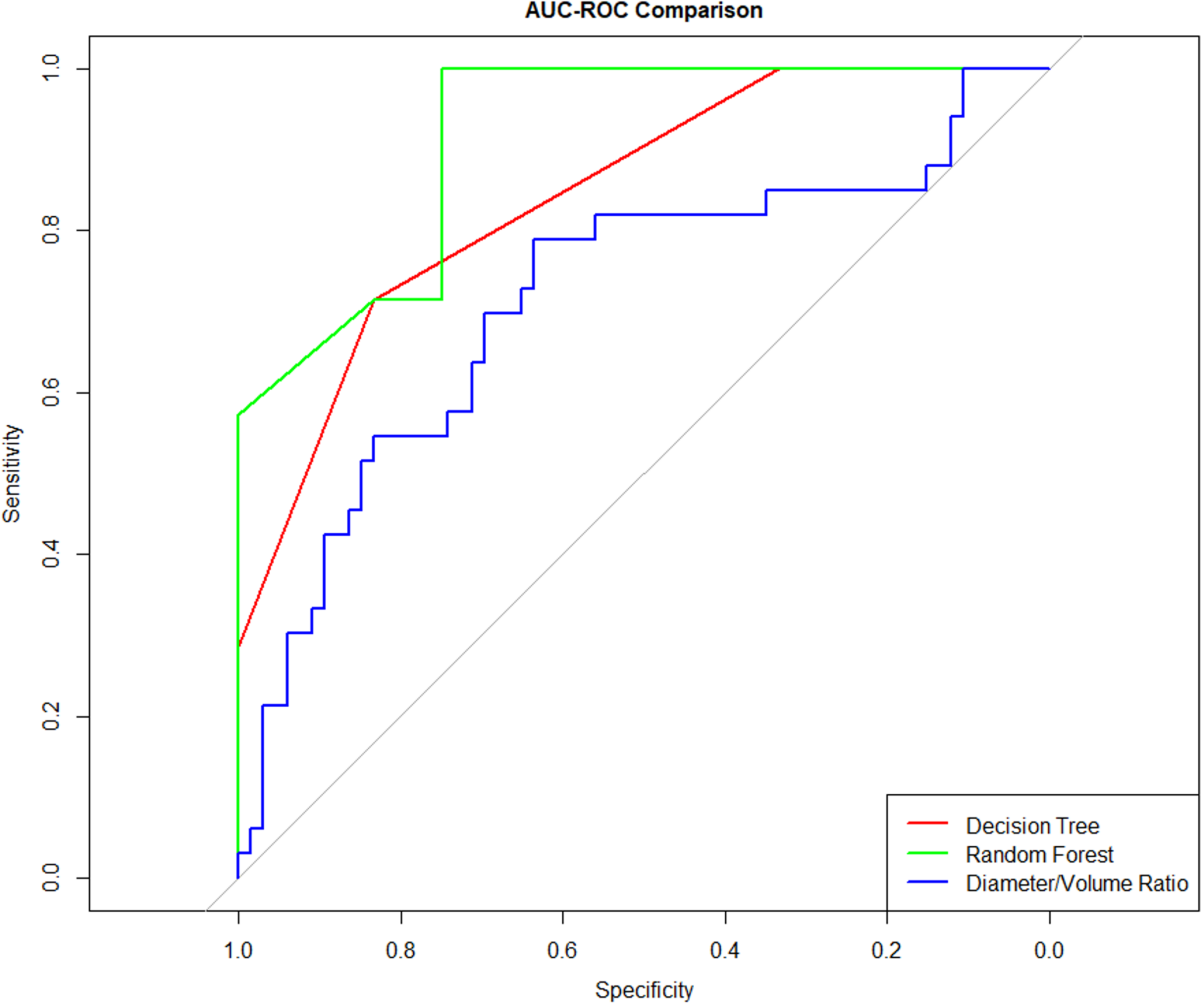
AUC comparison among predictors

Decision tree (Fig. 3) and random forest (Fig. 4) models were built to predict the probability of treatment failure. Decision trees were constructed to split the data based on variables such as metastasis volume and anatomical location, as shown in the tree diagram. The tree model reached nodes with different predicted probabilities of failure, indicating that lower final tumor volumes and specific brain locations are associated with better outcomes. The decision tree’s performance demonstrated an area under the curve (AUC) of 0.85 (Fig. 5).

The random forest model enhanced this analysis by aggregating multiple decision trees, thus reducing overfitting and improving prediction stability. The feature importance plots revealed that key predictors such as final metastasis volume, final diameter, and age played a significant role in the classification process. This model achieved a higher AUC of 0.92, indicating superior performance in comparison to the decision tree model.

In addition to the decision tree and random forest models, the diameter-to-volume ratio was also evaluated for its predictive performance. The area under the curve (AUC) for this feature was 0.72, indicating moderate predictive ability. ROC curves were fitted to compare the models’ performances.

## Discussion

This article demonstrated that an artificial intelligence model (random forest) presented an excellent predictive ability for stereotactic radiosurgery success/failure in a population with NSCLC brain metastases. This predictive ability was superior to a decision tree or a simple diameter-to-volume ratio.

Stereotactic radiosurgery (SRS) is an effective treatment option for brain metastases from non-small cell lung cancer (NSCLC), particularly for patients with a limited number of metastases. According to the ASCO-SNO-ASTRO guidelines, SRS is recommended for patients with one to four smaller brain metastases (< 4 cm) due to its association with less cognitive deterioration compared to whole-brain radiation therapy (WBRT). The guidelines emphasize that SRS is suitable for patients who prioritize cognitive function over intracranial control, as survival outcomes appear comparable between SRS and WBRT for this patient group [4].

Clinical studies support the effectiveness of SRS in achieving high local control rates with low morbidity [13, 14]. For instance, a retrospective analysis reported a 1-year local control rate of 78.7% for NSCLC brain metastases treated with SRS [15]. Another study found that patients with fewer than four brain metastases treated with SRS had longer survival compared to those treated with WBRT, even after adjusting for confounding factors [16]. Selection of patients most prone to treatment success remains, however, a crucial matter.

Artificial intelligence has helped improve outcome prediction in patients with brain metastases [17–19]. In particular, the increased robustness of random forest, through techniques such as bootstrapping and node-splitting on randomly selected features [20–22], led to more reliable predictions. In our study, it provided the best AUC.

Regarding the diameter-to-volume ratio, this metric shows that the ratio can distinguish between outcomes better than random guessing, it falls short compared to the more sophisticated models. This result suggests that although the diameter-to-volume ratio captures some relevant information about tumor progression, it lacks the complexity and depth of decision tree and random forest models, which leverage multiple features and non-linear relationships to improve prediction accuracy.

In the ROC curve comparison, the diameter-to-volume ratio’s curve lies below both the decision tree and random forest models, reflecting its lower predictive capability. Nonetheless, it serves as a valuable single-feature benchmark, illustrating that more complex models are required to achieve higher classification performance in this dataset. This analysis highlights the advantage of using machine learning models that integrate multiple predictors over relying on isolated features.

### Strenghts and limitations

This study presents some limitations. Data collection was retrospective, and sample size is not large. Furthermore, indication of stereotactic radiosurgery, although supported by guidelines, is not uniform among practicioners, and there might have been some selection bias. However, the study demonstrated an excellent predictive ability with real-world data.

## Conclusion

A random forest based artificial intelligence model presented an excellent predictive ability for stereotactic radiosurgery success/failure in a population with NSCLC brain metastases, with an area under curve of 0.92. This predictive ability was superior to a decision tree or a simple diameter-to-volume ratio.

## Data Availability

Data is available upon reasonable request.
